# Emerging role of C5aR2: novel insights into the regulation of uterine immune cells during pregnancy

**DOI:** 10.3389/fimmu.2024.1411315

**Published:** 2024-06-20

**Authors:** Fenna Froehlich, Konstanze Landerholm, Johanna Neeb, Ann-Kathrin Meß, Daniel Leonard Seiler, Tamara Tilburgs, Christian Marcel Karsten

**Affiliations:** ^1^ Institute for Systemic Inflammation Research (ISEF), University of Lübeck, Luebeck, Germany; ^2^ Division of Immunobiology, Cincinnati Children’s Hospital Medical Center, Cincinnati, OH, United States; ^3^ Department of Pediatrics, University of Cincinnati College of Medicine, Cincinnati, OH, United States

**Keywords:** anaphylatoxins, C5aR2, pregnancy, uterine NK cells, uterine DCs, INF-γ

## Abstract

Pregnancy is a fascinating immunological phenomenon because it allows allogeneic fetal and placental tissues to survive inside the mother. As a component of innate immunity with high inflammatory potential, the complement system must be tightly regulated during pregnancy. Dysregulation of the complement system plays a role in pregnancy complications including pre-eclampsia and intrauterine growth restriction. Complement components are also used as biomarkers for pregnancy complications. However, the mechanisms of detrimental role of complement in pregnancy is poorly understood. C5a is the most potent anaphylatoxin and generates multiple immune reactions via two transmembrane receptors, C5aR1 and C5aR2. C5aR1 is pro-inflammatory, but the role of C5aR2 remains largely elusive. Interestingly, murine NK cells have been shown to express C5aR2 without the usual co-expression of C5aR1. Furthermore, C5aR2 appears to regulate IFN-γ production by NK cells *in vitro*. As IFN-γ produced by uterine NK cells is one of the major factors for the successful development of a vital pregnancy, we investigated the role anaphylatoxin C5a and its receptors in the establishment of pregnancy and the regulation of uterine NK cells by examinations of murine C*5ar2^–/–^* pregnancies and human placental samples. C*5ar2^–/–^* mice have significantly reduced numbers of implantation sites and a maternal C5aR2 deficiency results in increased IL-12, IL-18 and IFN-γ mRNA expression as well as reduced uNK cell infiltration at the maternal-fetal interface. Human decidual leukocytes have similar C5a receptor expression patterns showing clinical relevance. In conclusion, this study identifies C5aR2 as a key contributor to dNK infiltration and pregnancy success.

## 1 Introduction

Pregnancy presents a complex immunological challenge because of the need to protect the immunologically foreign fetus from the maternal immune system without increasing her susceptibility to pathogens (1). As a result, the maternal immune system undergoes innumerable adaptions that allow implantation and placentation (2, 3). An ancient and highly conserved component of innate immunity is the complement system, which provides a first line of defense against pathogens (4). Complement activation leads to a self-amplifying cascade that ultimately results in, among other things (opsonization and formation of the membrane attack complex, MAC), the release of highly pro-inflammatory anaphylatoxins, of which C5a is the most potent (5, 6). Due to its high inflammatory potential, pregnancy requires tight regulation of the complement system, as excessive activation has been implicated in the pathogenesis of pregnancy complications including early pregnancy loss, fetal growth restriction, hypertensive disorders of pregnancy, and preterm birth (7). However, uncomplicated pregnancy is also characterized by systemic activation of the complement system (8), suggesting a physiological role during pregnancy. Previous studies have shown that complement activation and function are involved in implantation, placental development, normal fetal development, and labor (9). To date, two receptors for C5a, C5aR1 (CD88) and C5aR2 (C5L2), have been identified (10, 11). While it is known that C5aR1 is a G-protein-coupled seven-transmembrane receptor that triggers pro-inflammatory processes such as chemotaxis, cytokine release and oxidative burst of immune cells upon C5a binding (12), the role of C5aR2 remains enigmatic (13, 14). C5aR2 lacks G-protein coupling and has therefore often been described as a decoy receptor that modulates C5aR1 signaling (15). Interestingly, however, studies using C5aR1 and C5aR2 reporter mice have shown that C5aR2, but not C5aR1, is expressed on murine NK cells, suggesting a specific function of C5aR2 on NK cells that is independent of C5aR1 (16, 17). Furthermore, a large body of recent research findings contradicts the decoy receptor hypothesis (18–20), which is supported by our findings that C5aR2 has been shown to be able to regulate the production of interferon-γ (IFN-γ) by peripheral natural killer (pNK) cells (17).

A distinct subset of NK cells are the uterine NK (uNK) cells, which constitute the largest leukocyte population in the murine and human decidua during early pregnancy. uNK cells play a unique role in placental development, placental immunity and pregnancy success (21, 22). UNK cells differ from blood pNK cells in size, function, and surface molecules (23). Murine uNK cells can be distinguished by their unique reactivity to Dolichus biflorus agglutinin (DBA), which is not found in other cell types or species (24). In contrast, human uNK cells – also called decidual NK (dNK) cells – are characterized by their CD56^bright^ CD16^-^ phenotype (25). Murine and human uNK cells have lower cytotoxicity than pNK cells (26–29) and are high producers of cytokines, chemokines, and angiogenic factors that mediate key physiological processes during placentation (30).

Although uNK cells are in direct contact with semi-allograft fetal trophoblast cells, they do not exert any cytolytic functions against these cells due to the expression of non-classical MHC-I molecules (31, 32). Nevertheless, uNK cells have important functions in protecting the fetus from viral or bacterial infections of the placenta via Killer Ig-Like Receptors (KIRs) or through the production of the antimicrobial peptide granulysin (22, 33). In addition to their protective role, uNK cells are critically involved in the establishment of normal implantation sites and in adequate remodeling of the decidual spiral arteries in mice, which is required for adequate blood flow within the placenta. This process is mainly mediated by uNK cell-derived IFN-γ, indicating the importance of uNK cell functions in the development of a vital pregnancy (34, 35). In addition to IFN-γ, human and mouse uNK cells produce a variety of other cytokines, growth, and angiogenic factors that support the developmental processes within the placenta, suggesting that sufficient uNK cell activation may be necessary to reduce the risk of pregnancy complications such as pre-eclampsia (30). Within the decidual environment, uNK cells interact with uterine dendritic cells (uDCs), which are capable of producing NK cell-activating factors such as IL-12, IL-15, and IL-18 (36). This crosstalk and direct cell-cell contact is required for proper uNK cell function, as inhibition of uDC recruitment to the pregnant uterus results in impaired uNK cell development, a reduction in their IFN-γ production, and structural abnormalities of the spiral arteries (37).

Based on previous findings (16, 17), we hypothesize that C5aR2 is a novel regulator of uNK cell function and plays an important role during pregnancy.

## 2 Materials and methods

### 2.1 Breeding analysis

Data from our internal breeding facility were analyzed for breeding efficiency of syngeneic matings of C57BL/6j wild type (WT x WT), C5aR2-deficient (*C5ar2^–/–^* x *C5ar2^–/–^*), and C5aR1-deficient (*C5ar1^–/–^* x *C5ar1^–/–^*) mice based on their litter size. Knock-out strains were based on the C57BL/6j background. Mouse breeding was performed and documented by trained personnel. Litters within one year were included in the analysis. Mice used for internal breeding were maintained in a pathogen-free environment with a 12-h:12-h light-dark cycle. The number of live-born pups and pups found dead shortly after parturition per litter was used to compare their breeding efficiency. Furthermore, the total number of healthy implantation sites (IS) in the harvested uteri of WT and *C5ar2^–/–^* x WT matings used for further experiments was analyzed.

### 2.2 Animals, mating procedure and organ harvest

C57BL/6j wild-type (WT), C5aR2 knock-out (*C5ar2^–/–^*), and tdTomato floxed C5aR2-knock-in (C5aR2 reporter) mice on C57BL/6j background were bred in the breeding facility of the University of Lübeck. All animals were used at 12–20 weeks of age, and the handling was performed according to the appropriate institutional and national guidelines. Mice were maintained in a pathogen-free environment with a 12-h:12-h light-dark cycle. To make females susceptible to mating taking advantage of the Whitten effect, some stray containing the male’s urine was placed in their cage three days before mating to induce estrus. Female mice of both genotypes were mated with WT males overnight, checked for a vaginal plug the next morning defined as gestation day (gd) 1 and terminated at gd 8 to gd 10 by cervical dislocation. The IS were harvested and placed in 6-well plates containing RPMI medium after removal of surrounding fat and vessels. Their number was documented, and photographs were taken. Isolated IS were stored on ice until further processing. The procedure was approved by the local authorities of the Animal Care and Use Committee (Ministerium für Energiewende, Landwirtschaft, Umwelt und ländliche Räume des Landes Schleswig-Holstein, Kiel, Germany). All experiments were performed by certified personnel. The mice were kindly provided by Jörg Köhl, Institute for Systemic Inflammation Research (ISEF), Luebeck.

### 2.3 Immunofluorescence staining and confocal microscopy

IS of gd 8 were dissected, frozen in liquid nitrogen and stored at -80°C until further use. Serial cryosections of 6 µm were made using the Leica Kryostat CM3050 S and fixed in acetone (JT Baker). They were stored at -20°C until further use. Before staining, sections were thawed, encircled using a DAKO liquid fat pen and washed three times in PBS. They were blocked for 15 minutes with F_c_-block (anti-CD16/CD32; dilution 1:100) for 15 minutes in a StainTray (eBioscience) and then washed again three times in PBS. The staining mastermix containing DAPI (Life Technologies; dilution 1:1000), PE-CF594-conjugated anti-CD11c antibody (BD Biosciences; dilution 1:100) or PE-conjugated anti-Thy1.2 (BD Pharmingen) and fluorescein-conjugated DBA (Vector laboratories; dilution 1:500) was applied and incubated for 45 minutes in the dark. The washing procedure was repeated, and coverslips (Glaswarenfabrik Karl Hecht GmbH und Co. KG) were placed on the sections using Fluoroshield mounting medium (Sigma-Aldrich).

Slides stained against CD11c, and DBA were viewed with the Keyence BZ 9000 und photographed with the BZ Viewer software. Image merges and overlays were created, and scales inserted using the BZ analyzer software or the FIJI software. Only every tenth section was used for analysis to avoid double counting of cells. At least three different sections per specimen and six specimens per genotype were analyzed for frequency, infiltration area and depth of DBA^+^ uNK cells using the FIJI software. The diameters and surface areas of DBA^+^ uNK cells were measured using the same software. A minimum of 50 centrally sectioned uNK cells per section, three sections per specimen, and six specimens per genotype were analyzed.

Sufficient image quality of the staining of Thy1.2 and DBA could only be achieved using the Confocal Laser-Scanning-Mikroscope FluoView V1000 by Olympus. Images were analyzed using the FIJI software. At least three different sections per specimen and five specimens per genotype were analyzed for frequency of Thy1.2^+^ and DBA^+^ uNK cells.

### 2.4 Flow cytometry

IS of WT and *C5ar2^–/–^* x WT matings were freshly dissected at gd 8, cleared from surrounding tissue, and transferred to 5 mL of RPMI medium. DNase and Liberase TL (both Hoffmann – La Roche AG) were added at concentrations of 0.5 mg/ml and 0.1 mg/ml, respectively. The tissue was minced and enzymatically digested for 45 minutes at 37°C on a waving shaker. The tissue was then forced through a 100-µm cell strainer, washed with PBS, and centrifuged. After a 15-minute blocking step with F_c_-block (anti-mouse CD16/CD32 antibody) and incubation with the fixable viability dye eFluor780 (Invitrogen; dilution 1:1000), cells from IS of WT and *C5ar2^–/–^* x WT matings were stained with the antibodies for the NK cell (**Table 1**) and the DC panel (**Table 2**), respectively, for 15 minutes at 4°C in the dark. Antibodies were diluted to the appropriate concentrations in PBS containing 1% BSA. After a washing step, cells were fixed, permeabilized with Cytofix/Cytoperm solution (BD Biosciences) and washed with PermWash buffer (BD Biosciences) according to the manufacturer’s instructions. Cells used for the NK cell panel were then incubated with fluorescein-conjugated DBA (Vector Laboratories) for 45 minutes and washed before analysis. Positive staining was identified by *Fluorescence-minus-one* (FMO) controls for each target. Data were acquired using the BD LSR II and analyzed using FlowJo software version 10.7.1.

**Table 1:** Anti-murine antibodies used for the analysis of murine NK cells.

Antigen	Target	Isotype	Manufacturer	Fluorochrome	Dilution
CD3	T cells	Rat IgG_2b_, κ	BioLegend	BV510	1:400
CD16/32	Fc-block	Rat IgG2a, λ	Invitrogen	–	1:300
CD45	30-F11	Rat IgG_2b_, κ	eBioscience	AF700	1:800
CD122	TM-β1	Rat IgG2a, κ	BD Biosciences	BV421	1:400
DBA	NK cells	–	Vector Laboratories	Fluorescein	1:500

**Table 2:** Anti-murine antibodies used for the analysis of murine dendritic cells.

Antigen	Target	Isotype	Manufacturer	Fluorochrome	Dilution
CD3e	T cells (dump channel)	Armenian Hamster IgG_1_, κ	BioLegend	FITC	1:400
CD16/32	Fc-block	Rat IgG2a, λ	Invitrogen	–	1:300
CD19	B cells (dump channel)	Rat Lewis IgG_2a_, κ	BD Pharmingen	FITC	1:400
CD49b	NK cells (dump channel)	Rat Lewis IgM, κ	BD Pharmingen	FITC	1:400
F4/80	Macrophages (dump channel)	Rat IgG2a, κ	Invitrogen	FITC	1:300
Ly6G	Neutrophils	Rat Lewis IgG_2a_, κ	BD Pharmingen	FITC	1:400
MHC-II	Dendritic cells	Armenian Hamster IgG_1_, λ2	BioLegend	PE-Cy7	1:500
CD11c	Dendritic cells	Rat IgG2a, κ	BD Biosciences	APC	1:400

### 2.5 Real-time-PCR

To isolate RNA from IS, the Qiagen RNeasy Mini Kit was used according to the manufacturer’s instructions. Tissue was homogenized in RLT buffer for approximately 30 seconds using the ULTRA-TURRAX T8 homogenizer prior to performing the protocol provided. For transcription of RNA into cDNA, the PrimeScript RT Reagent Kit with gDNA Eraser was used according to the manufacturer’s instructions. Real-time quantitative polymerase chain reaction (qPCR) was performed targeting mRNAs transcribed from *interleukin 15* (*Il15*), *interleukin 18* (*Il18*), *interferon gamma* (*Ifng*), and *transforming growth factor beta 1* (*Tgfb1*). *β-Actin (Actb)* was used as a housekeeping gene to normalize the expression of the target genes. The following primers used for qPCR were purchase from Eurofins Genomics Germany GmbH (Ebersberg, Germany): IFN-γ forward 5’-CCA TCC TTT TGC CAG TTC CTC-3’, IFN-γ reverse 5’-ATG AAC GCT ACA CAC TGC ATC-3’, IL-15 forward 5’-ACA TCC ATC TCG TGC TAC TTG T-3’, IL-15 reverse 5’-GCC TCT GTT TTA GGG AGA CCT-3’, TGF-β1 forward 5’-AGC TGG TGA AAC GGA AGC G-3’, and TGF-β1 reverse 5’-GCG AGC CTT AGT TTG GAC AGG-3’. The primers IL-18 forward 5’-GTA TTA CTG CGG TTG TAC AGT G-3’ and IL-18 reverse 5’-GCG AGC CTT AGT TTG GAC AGG-3’ were obtained from MWG-Biotech AG (Ebersberg, Germany). The primers β-Actin forward 5’-AAA TAG CAG CCT GGA TAG CAA C-3’ and β-Actin reverse 5’-GCA CCA CAC CTT CTA CAA TGA G-3’ were used for the housekeeping gene (Invitrogen, Thermo Fisher Scientific, Waltham, Massachusetts, USA). The Bio-Rad iQ™ SYBR® Green Supermix was used for dye-based real-time quantification according to the manufacturer’s instructions. For the quantification of mRNA levels of *placental growth factor* (*Plgf*), a TaqMan probe assay (assay ID qMmuCIP0033405, purchased from Bio-Rad) was used with *glyceraldehyde-3-phosphate dehydrogenase* (*Gapdh*) serving as a housekeeping gene (assay ID: Mm99999915_g1, purchased from Applied Biosystems). For the assay, the Taqman Gene Expression Mastermix (Applied Biosystems) was used according to the manufacturer’s instructions. Samples were pipetted in triplicates. Afterwards, thermocycling protocols were performed using the 7900 HT Fast Real Time PCR System.

To calculate the induced fold changes, the ΔΔCt method by Livak and Schmittgen was applied to the samples (38). The values of the WT x WT matings served as reference controls for the calculation of fold changes within the samples of *C5ar2^–/–^* x WT matings. Statistical analysis of the data was performed by comparing the ΔCt values of the respective targets.

### 2.6 Analysis of inner lumen:outer diameter ratio

IS of gd 10 were dissected, frozen in liquid nitrogen and stored at -80°C until further use. Serial cryosections of 6 µm were generated using the Leica Kryostat CM3050 S and fixed in acetone (JT Baker). They were stored at -20°C until further use. Prior to staining, the sections were thawed, encircled using a DAKO liquid fat pen and washed three times in PBS. They were stained with Epredia™ Shandon™ Kwik-Diff™ Stain Kit according to the manufacturer’s instructions. Microscopic images were taken and the inner lumen:outer diameter ratio was calculated as described (39).

### 2.7 Preparation of human blood and placentas and flow cytometry analysis

In cooperation with the Department of Gynecology and Obstetrics of the University Hospital Schleswig-Holstein, Campus Lübeck, woman giving birth were recruited to provide their placenta and a blood sample after birth for the examination of the leukocytes contained therein. Samples from 8 term pregnancies were included in the study. The mothers were between 23 and 42 years old. In 7 pregnancies, the mode of delivery was caesarean section due to maternal risk factors such as previous caesarean section or maternal cardiopulmonary disease. One birth was by vacuum extraction. The gestational age at birth was between 34 + 1 and 40 + 2 weeks. 3 women were diagnosed with gestational diabetes. One mother had pre-existing arterial hypertension. None of the women were diagnosed with pre-eclampsia, HELLP syndrome or other pregnancy-related hypertensive disorders. The placenta was stored refrigerated until processing. For isolation of the *decidua basalis*, the placenta was positioned with the maternal side up and individual pieces were cut from the tissue. The placental villi were carefully removed until only the thin mucosal layer remained. This was then transferred into a 50-mL tube filled with PBS. For isolation of the *decidua parietalis*, the placenta was positioned with the fetal side facing up and the amnion was carefully detached. Then, a piece of the remaining membranes (chorion and *decidua parietalis*) was cut off from the rest of the placenta, and the *decidua parietalis* was carefully detached using sterile forceps. The decidual tissue was washed several times with PBS until no more blood was macroscopically detectable in the liquid phase. The tissue was then minced using dissecting scissors and washed with PBS as described before. Centrifugation was performed and the supernatant was discarded. RPMI medium containing DNase I (Hoffmann – La Roche, 0.5 mg/ml) and collagenase IV (Sigma-Aldrich, 1 mg/ml) was added. The tissue was incubated with the added enzymes in a shaking water bath at a temperature of 37°C for 90 minutes and then filtered using a cell dissociation sieve (“cell dissociation sieve - tissue grinder kit”). The solubilized cells were then filtered sequentially through cell sieves with a respective pore size of first 100 µm and then 40 µm. Samples were then centrifuged and resuspended in 20 ml RPMI and then layered over 10 ml of Histopaque-1077 (Sigma-Aldrich) in a new tube. This was followed by density gradient centrifugation. Mononuclear cells were isolated from the cell layer in the middle and washed twice with PBS. After leukocyte isolation was completed, cells were stained for flow cytometric analysis. Because CD16 was also stained as a marker, blockade of Fc receptors (CD32/CD16) was omitted. Cells were incubated with 1:1000 diluted eFluor780 fixable viability dye. After a brief centrifugation, cells were incubated with the antibodies for extracellular staining for 15 minutes (**Table 3**). After another washing step, cells were fixed and permeabilized with Cytofix/Cytoperm solution (BD Biosciences) for intracellular staining and then washed with PermWash buffer (BD Biosciences) according to the manufacturer’s instructions. This was followed by a 45-minute incubation with antibodies used for intracellular staining (Anti-C5aR1 and C5aR2, **Table 3**). After another wash step, samples were resuspended in 350 µL PBS/1%BSA and transferred to FACS tubes. Mononuclear cells from peripheral blood samples of the same donors were isolated by density gradient centrifugation and then washed and stained according to the same protocol. Samples were analyzed using the BD LSRII and gated using FlowJo software version 10.7.1. Gating was verified using fluorescence-minus-one (FMO) controls and isotype controls for C5aR1 and C5aR2.

**Table 3:** Anti-human antibodies used for the analysis of human decidual and peripheral leukocytes.

Antigen	Target	Isotype	Manufacturer	Fluorochrome	Dilution
CD3	T cells	Mouse IgG_1_, κ	BioLegend	PerCP	1:400
CD16	NK cells	Mouse IgG_1_, κ	BioLegend	PE-Cy7	1:200
CD45	Leukocytes	Mouse IgG_2a_, κ	Invitrogen	Superbright 780	1:300
CD56	NK cells	Mouse IgG_1_, κ	BioLegend	BV711	1:100
CD88 (C5aR1)	C5aR1+ cells	Mouse IgG_2a_, κ	BioLegend	APC	1:300
C5L2 (C5aR2)	C5aR2+ cells	Mouse IgG_2a_, κ	BioLegend	PE	1:400

### 2.8 Statistical analysis

Data was analyzed using GraphPad Prism version 8.0.1. Breeding data is shown as box-whisker-plots indicating mean, quartiles, and range of the data sets. RT-PCR data is expressed as mean ± SEM. Data on murine uterine NK cells and dendritic cells as well as human decidual cells are given as mean ± SEM. We tested the data sets for normal distribution using the Shapiro-Wilk test and used the Mann-Whitney U-test or the unpaired T-test depending on the result. P<0.05 was considered statistically significant.

### 2.9 Study approval

Animal experiments were approved by the local authorities of the Animal Care and Committee (Ministerium für Energiewende, Landwirtschaft, Umwelt und ländliche Räume des Landes Schleswig-Holstein, Kiel, Germany). The use of human material was approved by the ethics committee of the University of Luebeck (Ethikkomission der Universität zu Lübeck).

## 3 Results

### 3.1 Maternal C5aR2-deficiency impairs breeding efficiency in mice

**Figure 1:**
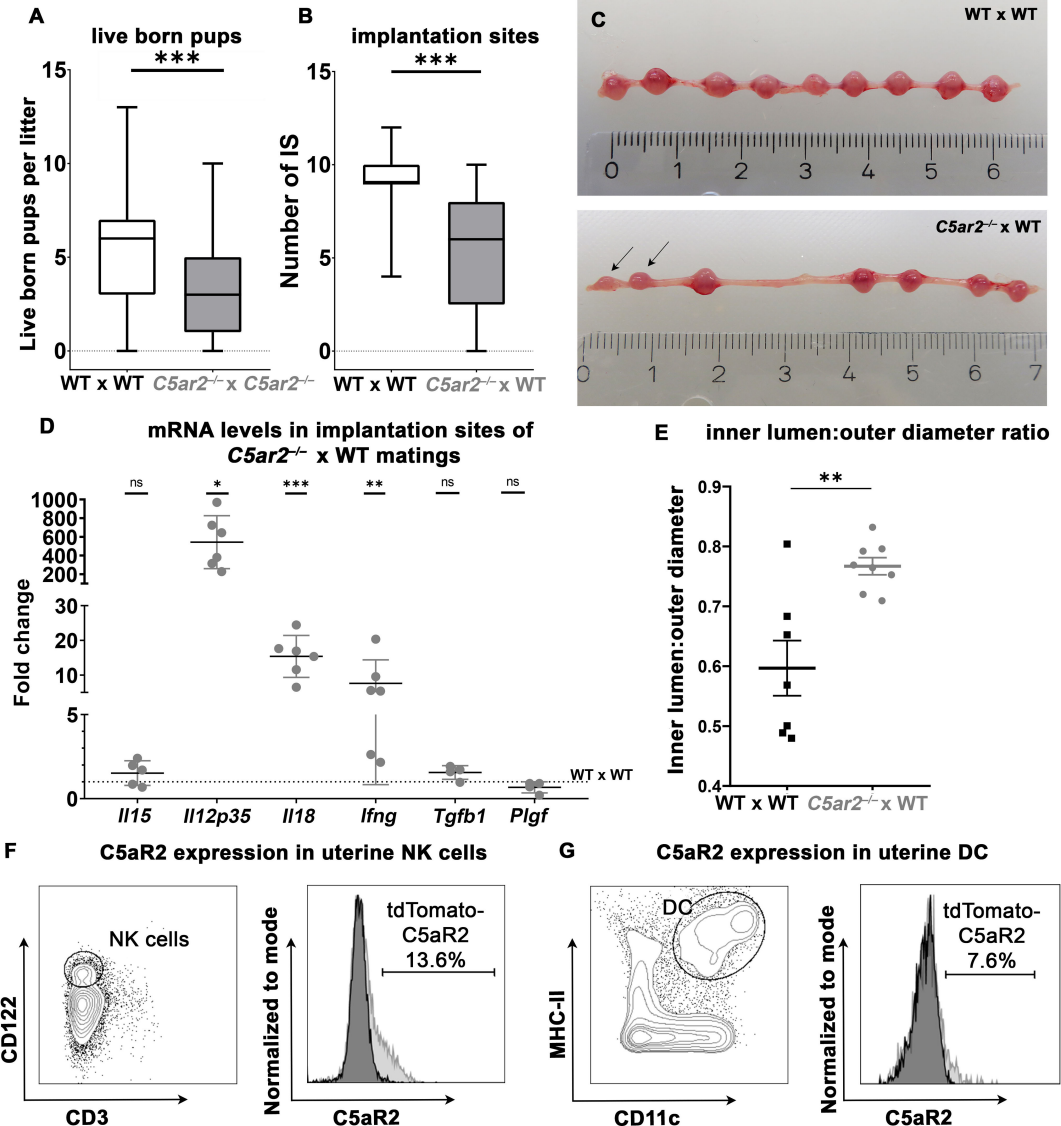
Breeding efficiency of WT x WT, *C5ar2^–/–^* x *C5ar2^–/–^*, and *C5ar2^–/–^* x WT matings. **(A)** Depicted is the number of live born pups per litter in WT x WT matings (n = 98) compared to *C5ar2^–/–^* x *C5ar2^–/–^* matings (n = 60). **(B)** Depicted is the number of healthy implantation sites (IS) at gd 8 to gd 10 in *C5ar2^–/–^* females mated with WT males (*C5ar2^–/–^* x WT, n = 27) compared with WT females mated with WT males (WT x WT, n = 17). Boxplots depict median and interquartile range. **(C)** Representative pictures of a pregnant uterus of a WT x WT (top panel) and *C5ar2^–/– -^* x WT (bottom panel) mating at gd 8. Incipient resorptions are marked with arrows. **(D)** mRNA levels of *Il15*, *Il12p35*, *Il18*, *Ifng*, *Tgfb1*, and *Plgf* in IS of *C5ar2^–/–^* x WT matings (n ≥ 4 normalized to mRNA levels in IS of WT x WT matings (n ≥ 5). Statistical analysis was performed by comparison of the delta ct values of each target. **(E)** Depicts the inner lumen to outer diameter ratio in IS of WT x WT and *C5ar2^–/–^* x WT matings at gd 10. **(F)** Representative FACS plots of CD3^-^ CD122^+^ uNK cells C5aR2-tdTomato signal of uNK cells from C5aR2-reporter x WT matings (light grey histogram) compared to WT x WT matings (dark grey histogram) at gd 8. **(G)** Representative plots of CD11c^+^ MHC-II^+^ uDCs and C5aR2-tdTomato signal in uDC cells from C5aR2-reporter x WT matings (light grey histogram) compared to WT x WT matings (dark grey histogram) at gd8. Lines represent mean and SEM. * p < 0.05; ** p < 0.01; *** p < 0.001; ns nonsignificant.

To investigate whether loss of the C5ar2 gene affects pregnancy outcome in mice, breeding data from our animal facility were analyzed for the number of live-born pups per litter in homozygous matings of C57BL/6j wild-type (WT) versus C57BL/6j *C5ar2^–/–^* mice. Over a period of one year, 98 litters of WT and 60 litters of *C5ar2^–/–^* pregnancies were documented. Interestingly, the litter size of WT matings averaged 5.3 (± 2.9) live-born pups, while a litter from *C5ar2^–/–^* matings averaged only 3.4 (± 2.6) live-born pups (**Figure 1A**). Notably, no abnormalities in reproductive efficiency were observed in *C5ar1^–/–^* mice compared to WT matings (**Supplementary Figure S1A**). In addition to litter size, the survival of individual offspring from each litter was also analyzed. We found that 20.5% of all pups from homozygous *C5ar2^–/–^* matings were found dead shortly after parturition (53 of 259 pups), compared to 10.3% of all pups in the WT group (61 of 590) and 6.8% in the *C5ar1^–/–^* group (13 of 191; **Supplementary Figure S1B**).

As we saw striking effects of C5aR2 deficiency on breeding efficiency in the data of our breeding facility, we wondered whether these effects were mechanistically related to C5aR2 deficiency in maternal tissue. To exclude potential effects caused by homozygous C5aR2 deficiency in the offspring, heterozygous matings with *C5ar2^–/–^* females and WT males were performed. A significant reduction in the number of healthy IS was observed in pregnant uteri of *C5ar2^–/–^* females mated with WT males (5.53 ± 3.17) compared to WT x WT matings (9.07 ± 1.64) (**Figures 1B, C**), demonstrating the detrimental effect of a maternal C5aR2 loss on pregnancy success.

### 3.2 Maternal C5aR2-deficiency alters mRNA levels of cytokines involved in NK cell and DC function in IS

To determine whether maternal C5aR2 deficiency affects cytokine expression in the IS, mRNA levels of pro- and anti-inflammatory cytokines and growth factors important for placentation were determined in the IS of WT x WT or *C5ar2^–/–^* x WT matings at gd 8 Transcripts of the pro-inflammatory cytokines *Il18*, *Ifng*, and *Il12p35* were increased statistically significant by 15-, 5-, and 543-fold, respectively, in the IS of *C5ar2^–/–^* x WT matings. The expression level of placental growth factor (*Plgf*) in the IS of *C5ar2^–/–^* x WT matings was nonsignificantly reduced by approximately 33%, compared to the IS of WT x WT matings. No significant difference in expression was observed for anti-inflammatory cytokine *Tgfb1* and the NK cell activating cytokine *Il15* (**Figure 1D**, **Supplementary Figure S2**). Thus, a maternal C5aR2 deficiency significantly increases pro-inflammatory factors ate the maternal-fetal interface.

### 3.3 Maternal C5aR2 deficiency causes hyperdilation of decidual arteries in IS

Successful pregnancy requires remodeling of the spiral arteries to enable adequate blood supply to the developing fetus. In murine pregnancy, this process is supported by IFN-γ production by uNK cells (34). As maternal C5aR2 deficiency caused elevated mRNA levels of *Ifng* as well as alterations in uNK cell number and infiltration depth, decidual arteries in IS of *C5ar2^–/–^* x WT matings and WT x WT matings were analyzed for vessel inner lumen to outer diameter ratios (lumen:diameter ratio), an indicator of vessel dilation (23). In the IS of *C5ar2^–/–^* x WT matings, spiral arteries were significantly more dilated (mean lumen:diameter ratio of 0.8) compared with arteries in the IS of WT x WT matings (mean lumen:diameter ratio of 0.6; **Figure 1E**). Representative pictures of the decidual vessels are shown in **Supplementary Figure S3**. Thus, a maternal C5aR2 deficiency influences placentation by causing excessive dilation of decidual arteries beyond the usual remodeling process during murine pregnancy.

### 3.4 C5aR2 is expressed in murine uNK cells and uDCs

Since the breeding efficiency and the cytokines involved in uNK cell and uDC crosstalk were strongly affected by maternal C5aR2 deficiency, C5aR2 expression on uNK and uDC was determined by flow cytometry in matings of female floxed tdTomato C5aR2 *knock-in* reporter mice (C5aR2 reporter) with male WT mice (C5aR2 reporter x WT matings). The C5aR2 reporter mice have a floxed gene encoding the reporter protein tdTomato, which is co-expressed with C5aR2 (Karsten et al., (17)). Phenotypically, these mice do not differ from their WT counterparts, and we did not observe any abnormalities during pregnancy (data not shown). Interestingly, in C5aR2 reporter x WT matings, a subset of uNK expressed tdTomato-C5aR2, with approximately 10–15% of CD3^-^CD122^+^ uNK cells staining positive for tdTomato-C5aR2 (**Figure 1F**). Approximately 3% of splenic NK cells from the same females showed C5aR2 expression (**Supplementary Figure S4A**). In lineage marker (CD3e, CD19, CD49b, Ly6G, F4/80)-negative MHCII^+^CD11c^+^ uDCs and splenic DCs, C5aR2 expression was found in approximately 7% of cells (**Figure 1G**, **Supplementary Figure 4B**). Thus, a subset of uNK and uDC express C5aR2 and are directly susceptible for complement signaling.

### 3.5 DBA^+^ uNK cell frequency, infiltration, and size are reduced in IS of *C5ar2^–/–^* mice

**Figure 2:**
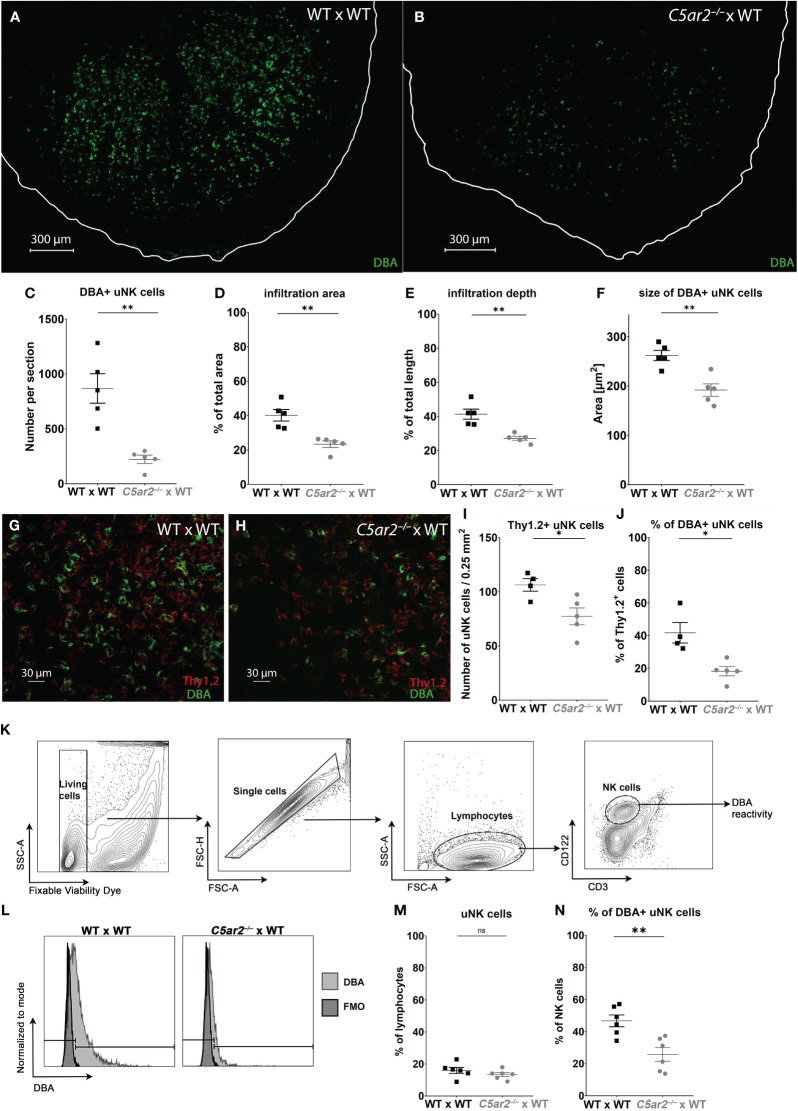
Uterine NK cell numbers and infiltration depth are reduced in the implantation sites of *C5ar2^–/–^* x WT matings. Representative picture the *decidua basalis* of a section of an IS of **(A)** WT x WT and **(B)** *C5ar2^–/–^* x WT matings stained with dolichos biflorus agglutinin (DBA) to identify DBA^+^ uNK cells at gd 8. Tissue borders are marked by the white line for orientation. Graphs depict **(C)** number, **(D)** infiltration area, **(E)** infiltration depth, **(F)** size of uNK cells in IS of WT x WT and *C5ar2^–/–^* x WT matings at gd 8. Representative pictures of Thy1.2 (red) and DBA (green) immunofluorescence staining in the *decidua basalis* of **(G)** WT x WT and **(H)** *C5ar2^–/–^* x WT matings at gd 8. Graphs depict **(I)** the number of Thy1.2^+^ uNK cells per 0.25 mm^2^ and **(J)** the proportion of DBA^+^ uNK cells to Th1.2^+^ uNK in the *decidua basalis* of WT x WT and *C5ar2^–/–^* x WT matings at gd 8. **(K)** Gating strategy used for the flow cytometric analysis of DBA reactivity of uNK cells in IS of WT x WT and *C5ar2^–/–^* x WT matings at gd 8. **(L)** Representative FACS plots of the DBA staining in CD3^-^ CD122^+^ uNK cells compared to FMO in IS of WT x WT and *C5ar2^–/–^* x WT matings at gd 8. Graphs depict **(M)** the share of CD3^-^ CD122^+^ uNK cells among all lymphocytes and **(N)** the share of DBA^+^ uNK cells as a percentage of all NK cells in IS of WT x WT and *C5ar2^–/–^* x WT matings. Graphs depict mean and SEM. * p < 0.05; ** p < 0.01; ns nonsignificant.

To gain insight into the role of C5aR2 in uNK infiltration and their activation at the maternal fetal interface, immunofluorescence staining with DBA was performed to analyze the uNK frequency and their infiltration depth (**Figures 2A, B**). In the IS of WT x WT matings, a mean frequency of 867.8 DBA^+^ uNK cells per tissue section was observed at gd 8 (**Figures 2A, C**). In contrast, a mean frequency of 222.7 DBA^+^ uNK cells per section was observed in the IS of *C5ar2^–/–^* x WT matings at gd 8 (**Figures 2B, C**). In addition, a significantly reduced infiltration area (**Figure 2D**) and depth (**Figure 2E**) of DBA^+^ uNK was observed in the IS of *C5ar2^–/–^* x WT matings compared to WT x WT matings. In the IS of WT x WT matings, DBA^+^ uNK cells infiltrated 40.15% of the total IS area, whereas in *C5ar2^–/–^* x WT matings, DBA+ uNK cells infiltrated only 23.36% of the IS area. Similarly, the mean infiltration depth in the IS of WT x WT matings was 41.33% of the total IS length, whereas a mean infiltration depth of 27.19% was observed in *C5ar2^–/–^* x WT matings.

Analysis of uNK cell size in the same images revealed a significant reduction in the IS of *C5ar2^–/–^* x WT matings compared to WT x WT matings (**Figure 2F**). Size is a measure of uNK cell maturation, with larger uNK cells being more mature (Felker and Croy (40)). Here, the DBA^+^ uNK cells of the IS of WT x WT matings measured 262.1 µm^2^, whereas in the IS of *C5ar2^–/–^* x WT matings we found significantly smaller uNK cells measuring 191.8 µm^2^ (**Figure 2F**).

### 3.6 Maternal C5aR2-deficiency shifts the distribution of the uNK cell population to a predominant DBA^-^ uNK cell phenotype

Since no lymphocyte population in any tissue outside the uterus or in virgin mice shows positive DBA reactivity, DBA is a good marker for uNK cells (41). However, there are also uNK cells that are negative for DBA. These uNK differ not only in their DBA reactivity but also in their function. While DBA^+^ uNK cells have been shown to produce more angiogenic factors such as PlGF, DBA^-^ uNK cells resemble pNK cells and mainly produce IFN-γ (42). To determine whether the DBA^-^ population was also affected by maternal C5aR2 deficiency, the proportion of Thy1.2+ uNK cells (a general marker for uNK cells including DBA^+^ and DBA^-^ uNK cell subsets) was determined (**Figures 2G-J**). The number of Thy1.2^+^ (DBA^+/-^) uNK cells per 0.25mm2 was significantly lower in the IS of *C5ar2^–/–^* x WT matings compared to the IS of WT x WT matings (**Figures 2G-I**). In addition, the percentage of DBA^+^ Thy1.2^+^ uNK cells was significantly lower in the IS of *C5ar2^–/–^* x WT matings (18%) compared to WT x WT matings (42%; **Figure 2J**). Flow cytometric analysis confirmed the decreased proportion of DBA^+^ uNK cells and increased proportion of DBA^-^ uNK cells in *C5ar2^–/–^* x WT matings (**Figures 2K-N**). Thus, maternal C5aR2 deficiency negatively affects the number of DBA^+^ uNK cells in the IS and shifts the uNK cell population toward a predominant DBA^-^ uNK cell phenotype.

### 3.7 Frequency of CD11c^+^ DCs and their colocalization with DBA^+^ uNK cells are diminished in IS of *C5ar2^–/–^* x WT matings

In addition to uNK cells, uDC play a key role in balancing maternal-fetal immune tolerance and immunity (43). Further uDC also have direct functions in attracting and activating uNK cells (44). To determine if maternal C5aR2 deficiency also affects the uDC population, we analyzed CD11c^+^ uDC frequency and co-localization of CD11c^+^ uDCs with DBA^+^ uNK cells in the IS of *C5ar2^–/–^* x WT matings compared to WT x WT matings at gd 8 using immunofluorescence microscopy.

**Figure 3:**
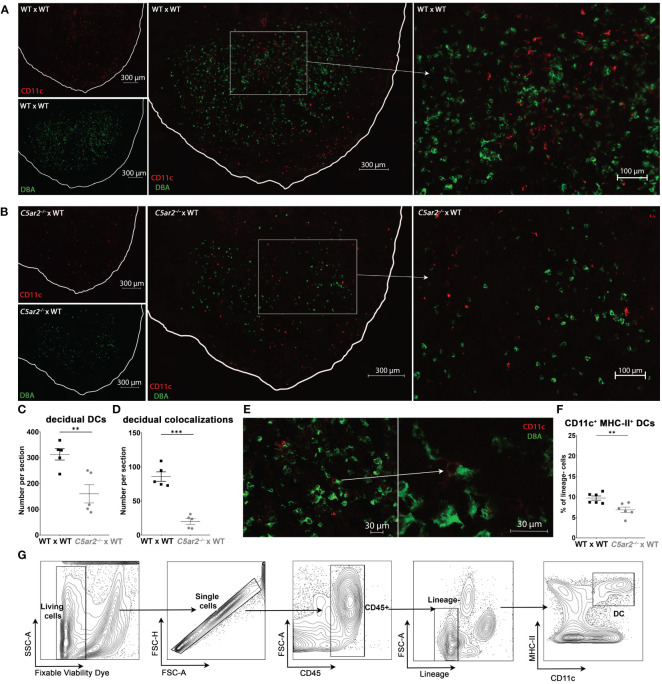
Uterine DCs and their co-localizations with DBA^+^ uNK cells in the IS of WT x WT and *C5ar2^–/–^* x WT matings. Representative pictures from the *decidua basalis* of IS sections from **(A)** WT x WT or **(B)** *C5ar2^–/–^* x WT matings after staining with DBA (green) and CD11c (red) to identify DBA^+^ uNK cells and CD11c^+^ uDC at gd 8. Tissue borders are marked by the white line for orientation. Left: CD11c^+^ cells (red, top panel) and DBA^+^ cells (green, bottom panel) in **(A)** WT x WT and **(B)** *C5ar2^–/–^* x WT matings. Middle panel shows the overlay of the two channels for CD11c and DBA in the overview of the decidual region in **(A)** WT x WT and **(B)** *C5ar2^–/–^* x WT matings. Enlarged details from the overview picture (marked with white rectangles) within the central zone of the *decidua basalis* of **(A)** WT x WT and **(B)** *C5ar2^–/–^* x WT matings are shown in the right panel. Graphs depict **(C)** the number of CD11c^+^ uDCs and **(D)** the number of DBA^+^ uNK cell-CD11c^+^ uDC co-localizations within the decidua of WT x WT and *C5ar2^–/–^* x WT matings at gd 8. **(E)** Representative co-localization of a DBA^+^ uNK cell and a CD11c^+^ uDC within the decidua of a WT x WT mating. **(F)** Frequency of CD11c^+^ MHC-II^+^ DCs among lineage-negative leukocytes in the IS of WT x WT and *C5ar2^–/–^* x WT matings identified by flow cytometric analysis at gd 8. **(G)** Gating strategy used for the flow cytometric analysis of uDCs in the IS of WT x WT and *C5ar2^–/–^* x WT matings at gd 8. Lineage markers used for the exclusion of T cells, B cells, NK cells, Neutrophils and Macrophages were CD3e, CD19, CD49b, Ly6G, F4/80. Graphs depict mean and SEM. ** p < 0.01; *** p < 0.001.

In the IS of *C5ar2^–/–^* x WT matings, the frequency of CD11c^+^ uDCs was significantly decreased compared with the frequency of CD11c^+^ DCs in WT x WT matings (**Figures 3A-C**). Next, the occurrence of CD11c^+^ uDC and DBA^+^ uNK cell co-localizations was determined by counting the number CD11c^+^ uDC with and without the proximity of DBA^+^ uNK cells (**Figures 3A, B**). Co-localizations of CD11c^+^ DCs with DBA^+^ uNK cells were significantly reduced in *C5ar2^–/–^* x WT matings (19.8 per section) compared with the IS of WT x WT matings (85.7 per section) (**Figure 3D**). The IS of WT x WT matings showed a high density of uDC and uNK cells and many co-localizations of both cell types in proximity in the central zone of the decidua basalis (**Figure 3A**). A typical uNK-uDC co-localization in the IS of WT x WT matings is shown in **Figure 3E**.

The reduction of CD11c^+^ uDC in the IS of *C5ar2^–/–^* x WT matings was also confirmed by flow cytometry (**Figures 3F, G**). In the IS of WT x WT matings, we found a mean of 9.72% CD11c^+^, MHC-II^+^ uDC gated within lineage-negative cells (CD3e^-^, CD19^-^, Ly6G^-^, F4/80^-^). In contrast, in IS of *C5ar2^–/–^* x WT matings a mean percentage of 6.86% uDC within the lineage-negative population was found (**Figure 3F**). In summary, our data show that maternal C5aR2 deficiency significantly reduced the number of CD11c^+^ DCs and their ability to colocalize with DBA^+^ uNK cells at the maternal-fetal interface, possibly contributing to reduced uNK cell activation by uDCs.

### 3.8 Human decidual NK cells, T cells and MΦ show different expression pattern of C5aR1 and C5aR2 compared to their peripheral blood counterparts

**Figure 4:**
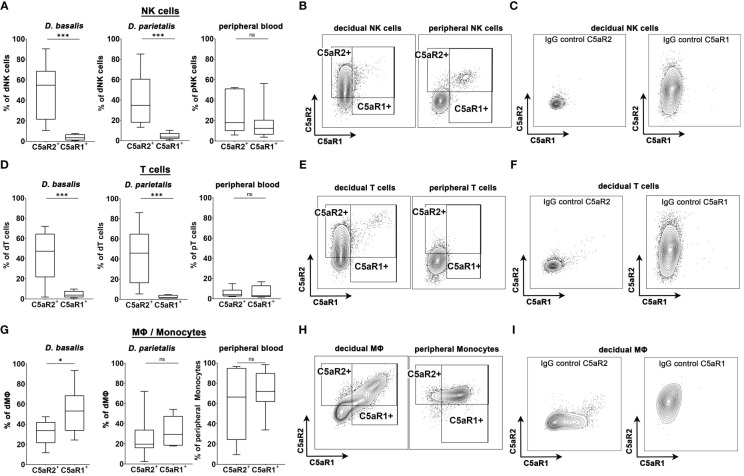
C5aR1 and C5aR2 expression patterns in human decidual cells. Graphs depict the share of C5aR1^+^ or C5aR2^+^ **(A)** NK cells, **(D)** T cells, and **(G)** MΦ/monocytes in the *decidua basalis (D. basalis)*, *decidua parietalis (D. parietalis)*, and peripheral blood of healthy pregnant woman at term. Representative FACS plots and gates for C5aR1 and C5aR2 expression of **(B)** decidual and peripheral NK cells, **(E)** decidual and peripheral T cells, and **(H)** decidual MΦ and peripheral monocytes are shown. Positive staining was identified by isotype controls for C5aR1 and C5aR2, respectively. Representative plots of the staining of the isotype controls in **(C)** decidual NK cells, **(F)** decidual T cells, and **(I)** decidual MΦ. For peripheral T cells one sample was identified as an outlier by Grubbs test and excluded from the analysis. Boxplots depict median and interquartile range. (n *_D.basalis_* =8, n *_D.parietalis_* =8, n _Peripheral blood_ =7). * p < 0.05; *** p < 0.001; ns nonsignificant.

We next investigated if human decidual immune populations (dNK cells, T cells and macrophages) express C5a receptors. Human decidual leukocytes obtained from placental samples of healthy term pregnancies were analyzed for their expression of C5a receptors by flow cytometry. The gating strategy is shown in **Supplementary Figure S5**. Peripheral blood leukocytes were isolated from the same donors and analyzed. In both the *decidua basalis (D. basalis)*, the maternal part of the placenta at the implantation site, and the *decidua parietalis (D. parietalis*), the maternal part of the placental membranes connected to the fetal chorion, we found a significant proportion of the dNK cells expressing C5aR2, whereas C5aR1 expression was almost completely absent (**Figures 4A-C**). This was in striking contrast to the expression pattern of C5aR2 and C5aR1 on peripheral blood NK cells (pNK), which were either double-negative or double-positive (**Figures 4A, B**).

Similar to the dNK cells, decidual T (dT) cells exclusively expressed C5aR2, whereas C5aR1 expression was virtually absent on these cells. C5a receptor expression was considerably lower in peripheral T (pT) cells compared to dT cells (**Figures 4D-F**). Interestingly, decidual MΦ (dMΦ) and peripheral blood monocytes (pM) both expressed C5aR1 and C5aR2 (**Figures 4G, I**). The frequency of C5aR1^+^ dMΦ in *D.basalis* was significantly higher than the frequency of C5aR2^+^ dMΦ, with an average of 53.2% cells being C5aR1^+^ and 31.9% of cells being C5aR2^+^ (**Figure 4H**). In *D. parietalis*, no significant difference was observed in the frequency of C5aR1^+^ and C5aR2^+^ dMΦ (**Figure 4G**). The pM showed high expression levels for both receptors, with mainly double-positive cells (**Figure 4H**). The selective expression of C5aR2 on decidual NK and T cells demonstrates the significance of the murine C5aR2 model for human pregnancy and leaves open many questions on how complement regulates decidual immune functions in healthy and complicated pregnancies.

## 4 Discussion

In this study, we highlight the importance of C5aR2 function in pregnancy. Pregnancy is a complex process requiring multiple adaptations of the maternal immune system to allow survival and protection of the semi-allogeneic fetus. Global maternal deficiency of the *C5ar2* gene severely impairs reproductive efficiency in mice (an effect not only observed at our institution but also reported by several collaborators working with these mice), suggesting a potential maternal dysregulation of the required adaptive mechanisms. Furthermore, we show in mice that the number of healthy IS in early gestation is significantly reduced in pregnancies with maternal C5aR2 deficiency. We identified increased mRNA levels of key pro-inflammatory cytokines involved uterine immune cell function in IS of *C5ar2* ^–/–^ pregnancies in combination with reduced dNK and uDC numbers, suggesting that the absence of C5aR2 signaling directly influences their function as well as infiltration into the maternal fetal interface. UNK cells are important regulators of spiral artery remodeling and trophoblast invasion. IFN-γ derived from uNK cells is the major factor inducing dilation of the spiral arteries during pregnancy (34). IFN-γ^–/–^ mice show decidual abnormalities, including narrowed spiral arteries. However, in the IS of *C5ar2^–/–^* x WT matings, mRNA expression levels of *Ifng* were greatly increased, combined with a massive hyperdilation of decidual vessels, resembling the findings of Senegas et al. in pregnant mice with *T. gondii* infection, in which increased IFN-γ levels and lower IL-15 levels caused hyperdilation of spiral arteries and fetal resorptions (39), suggesting similar mechanisms leading to pregnancy failure in these cases. C5aR2 may be directly involved in the mechanism of increasing intrauterine IFN-γ levels, as it is already known to increase IFN-γ production by pNK cells stimulated by IL-18 and IL-12 *in vitro* (17).

There are two major subsets of murine uNK cells that differ primarily in their reactivity to DBA (24), with the DBA^+^ subset producing mainly angiogenic factors such as PlGF, while the DBA- subset secretes mainly IFN-γ (42). The reduced absolute frequency of uNK cells in the IS of *C5ar2^–/–^* pregnancies was accompanied by a phenotypic shift toward the more IFN-γ-producing DBA- subset, complementing the reduced *Plgf* and concomitantly increased *Ifng* transcript levels. In addition, the increased intrauterine *Ifng* expression of pregnant *C5ar2^–/–^* females may be related to a dysregulation of the expression of the synergistic IFN-γ inducing factors IL-18 and IL-12. Both cytokines are produced by uDCs, which have important functions in mediating immunity and tolerance during pregnancy (45). Throughout pregnancy, there is reciprocal crosstalk between uDCs and uNK cells within the decidua, either indirectly through cytokine secretion or directly through cell-to-cell contact (44). In addition to altered mRNA levels of key cytokines, we also detected decreased numbers of uNK cells, uDCs, and uNK-uDC co-localizations in the IS of *C5ar2^–/–^* x WT matings. The reduced frequency of cell co-localization in combination with the altered cytokine mRNA levels indicates a severe dysregulation of NK-DC interaction at the maternal-fetal interface in *C5ar2^–/–^* x WT matings. Previous studies have shown that the absence of DCs in the murine IS results in decreased levels of IL-15 and IL-12 *in utero*, leading to reduced abundance, size, and IFN-γ expression of uNK cells (37). However, in *C5ar2^–/–^* x WT matings, the reduced frequency of uDCs was associated with similar levels of *Il15* transcripts but a massive increase in *Il12p35*, *Il18*, and *Ifng* expression within the IS. Possible explanations might be an overcompensation of the lowered cell number, but also a direct involvement of C5aR2 in the regulation of the expression of these cytokines by uNK cells and uDCs.

Not only the frequency, but also the size, infiltration depth and phenotype of uNK cells were affected by maternal C5aR2 deficiency. Normally, uNK cells have large diameters because they contain large amounts of granules despite their low cytotoxicity (46). Decreased cell size is indicative of reduced maturation (37, 40). *In utero*, DCs are the major source for IL-15 being the key cytokine for NK cell development (47). As absence of DCs as causes decreased uNK cell number and size (37), we propose impaired uNK-uDC crosstalk as the likely major cause of the altered uNK cells abundance, phenotype, and function in the IS of *C5ar2^–/–^* x WT matings. It has been shown that depletion of PlGF also leads to morphometric anomalies of the IS as well as an increased frequency of immature uNK cells (48). Accordingly, the reduced levels of *Plgf* transcripts in the IS of *C5ar2^–/–^* x WT matings could be involved in the development of the unfavorable environmental conditions.

In murine IS, uNK cells are restricted to the mesometrial site of the uterus. From gd 5 to gd 11, uNK cells start to proliferate, gain increasing numbers of cytoplasmic granules, and form a transient lymphoid structure known as the mesometrial lymphoid aggregate of pregnancy (MLAp; Peel (49); Paffaro et al., (24)). There is evidence for the recruitment of early NK cell precursors from the blood (50, 51), but also for the invasion of mature NK cells from the periphery (52). In mice, uNK cells initially accumulate in the *D. basalis* (around gd 6.5 (53);, but subsequently settle near by the spiral arteries (between gd 8.5 and gd 13.5) and begin to cooperate with the invading trophoblast cells to ensure correct remodeling of the arteries (21, 54). However, in murine pregnancy, the arterial media is mainly modified as a result of direct infiltration of uNK cells (54). The strong reduction in the infiltration of uNK cells into the decidua of the IS of *C5ar2^–/–^* females, indicates an impaired recruitment and infiltration process.

Using the well-established C5aR2-reporter mice (Karsten et al., (17)), we detected C5aR2 expression in uterine NK cells and DCs. However, not the entirety of uNK and uDC was positive for C5aR2, but only subsets of the cell types. This may indicate that not only the direct effects of the receptor on the NK cells and DCs but also effects caused by C5aR2 deficiency on other cell types interacting and influencing NK and DC function might be responsible for the effects seen in *C5ar2^–/–^* mice. Likewise, the C5aR2^+^ cells could be of special functional importance. It is also conceivable that the cells require C5aR2 for their functionality during their development. In order to differentiate between these possible explanations, studies using mice with cell specific C5aR2 deficiency are needed to elucidate the mechanistic process causing the distinct changes in cell phenotype and function as well as the dramatically impaired reproductive efficiency in C5aR2 deficient mice.

Peripheral human NK cells intracellularly express both receptors for C5a under steady-state conditions (55). Interestingly, our findings showed that a significant proportion of dNK cells expressed C5aR2, whereas C5aR1 expression was not detected. In contrast, pNK cells were either double-negative or double-positive for C5a receptors. This marked difference in C5a receptor expression on pNK and dNK suggests different modes of receptor function in the different NK cell types. Human dNK cells are essential regulators of implantation, trophoblast invasion and spiral artery remodeling and thus contribute critically to pregnancy success (56). Alterations in dNK cell number have been found in patients with pregnancy complications such as pre-eclampsia or intrauterine growth restriction associated with impaired placentation process (57). In particular pre-eclampsia is a severe condition resulting of incomplete trophoblast invasion and spiral artery remodeling with defects in NK cell crosstalk likely to be etiologically involved (56). However, both pre-eclampsia as well as its variant HELLP Syndrome (Hemolysis, Elevated Liver enzymes, Low Platelets) are associated with increased complement activation (58, 59). In placentas from pre-eclampsia patients, elevated C5a deposition in macrophages and increased C5aR1 expression on trophoblast cells as well as increased maternal serum C5a levels have been found (60). In HELLP syndrome, elevated C5a and C5b-9 levels are typically seen, and the condition is associated with mutations in complement genes (61). There is evidence that blocking C5 with eculizumab may be beneficial for pre-eclamptic patients (59). In one case report, treatment with eculizumab prolonged pregnancy and reduced symptoms in a HELLP patient (62). Collectively, these findings illustrate the need to control C5a-mediated inflammatory effects not during healthy pregnancy and in pregnancy complications.

C5aR2 may have a specific function in uNK cells in mice and humans, separate from C5aR1 and other cell types (18, 63). Complement components can be produced locally or intracellularly, influencing local complement activation and normal cell functions (64–67). Within cells, the function of C5a may differ from its classical chemoattractant properties. C5a produced by immune cells *in utero*, like uNK cells and uDCs, may contribute to spiral artery remodeling and trophoblast invasion, crucial for successful pregnancy. C5aR2 is mainly expressed intracellularly, including in decidual immune cells, suggesting its potential effect. Further studies are needed to understand the precise mechanisms of C5aR2 function in pregnancy.

In conclusion, our study demonstrates an essential role for C5aR2 function for a healthy pregnancy outcome. Maternal C5aR2 deficiency in mice causes severe impairments in reproductive efficiency, with reduced numbers of healthy implantation sites and altered mRNA levels of key cytokines involved in NK cell and DC function. These changes are accompanied by a dysregulation of uNK cell uDC crosstalk and a shift in uNK cell phenotype toward increased production of IFN-γ. These findings suggest that C5aR2 plays a critical role in immune system adaptations during pregnancy and may be a crucial target for improving pregnancy outcomes and reducing pregnancy complications.

## Data availability statement

The raw data supporting the conclusions of this article will be made available by the authors, without undue reservation.

## Ethics statement

The studies involving humans were approved by Ethikkomission der Universität zu Lübeck. The studies were conducted in accordance with the local legislation and institutional requirements. The participants provided their written informed consent to participate in this study. The animal study was approved by Ministerium für Energiewende, Landwirtschaft, 110 Umwelt und ländliche Räume des Landes Schleswig-Holstein, Kiel, Germany. The study was conducted in accordance with the local legislation and institutional requirements.

## Author contributions

FF: Writing – review & editing, Writing – original draft, Visualization, Methodology, Investigation, Formal analysis, Data curation. KL: Writing – original draft, Investigation. JN: Writing – original draft, Investigation. A-KM: Writing – original draft, Investigation. DS: Writing – review & editing, Writing – original draft, Validation, Supervision. TT: Writing – review & editing, Writing – original draft, Validation, Supervision, Methodology. CK: Data curation, Conceptualization, Writing – review & editing, Writing – original draft, Validation, Supervision, Resources, Project administration, Methodology, Funding acquisition.
