# Supplementary material for: Emerging role of C5aR2: novel insights into the regulation of uterine immune cells during pregnancy
Source: Front Immunol. 2024 Jun 20;15:1411315. doi: 10.3389/fimmu.2024.1411315 (PMC11229525; doi:10.3389/fimmu.2024.1411315)
Supplement: Supplementary file 1 [file DataSheet_1.docx]

Supplementary Material

Emerging role of C5aR2: novel insights into the regulation of uterine immune cells during pregnancy

**Fenna Froehlich^1^, Konstanze Landerholm^1^, Ann-Kathrin Meß^1^, Daniel Leonard Seiler^1^, Jörg Köhl^1,2^, Tamara Tilburgs^2,3^, Christian Marcel Karsten^1*^**

^1^Institute for Systemic Inflammation Research (ISEF), University of Lübeck, Germany

^2^Division of Immunobiology, Cincinnati Children’s Hospital medical Center, University of Cincinnati College of Medicine, Cincinnati, Ohio, USA

^3^Department of Pediatrics, University of Cincinnati College of Medicine, Cincinnati, Ohio, USA

*** Correspondence:**

Corresponding author: Christian Marcel Karsten
Adress: Institute for Systemic Inflammation Research (ISEF), University of Lübeck, Ratzeburger Allee 160, 23562 Lübeck

Email: [Christian.karsten@uksh.de](mailto:Christian.karsten@uksh.de)

Phone: +49 451 3101 8930

# Supplementary Data

Supplementary Material should be uploaded separately on submission. Please include any supplementary data, figures and/or tables.

Supplementary material is not typeset so please ensure that all information is clearly presented, the appropriate caption is included in the file and not in the manuscript, and that the style conforms to the rest of the article.

# Supplementary Figures and Tables

For more information on Supplementary Material and for details on the different file types accepted, please see [here](https://www.frontiersin.org/guidelines/author-guidelines#supplementary-material).

## Supplementary Figures

**
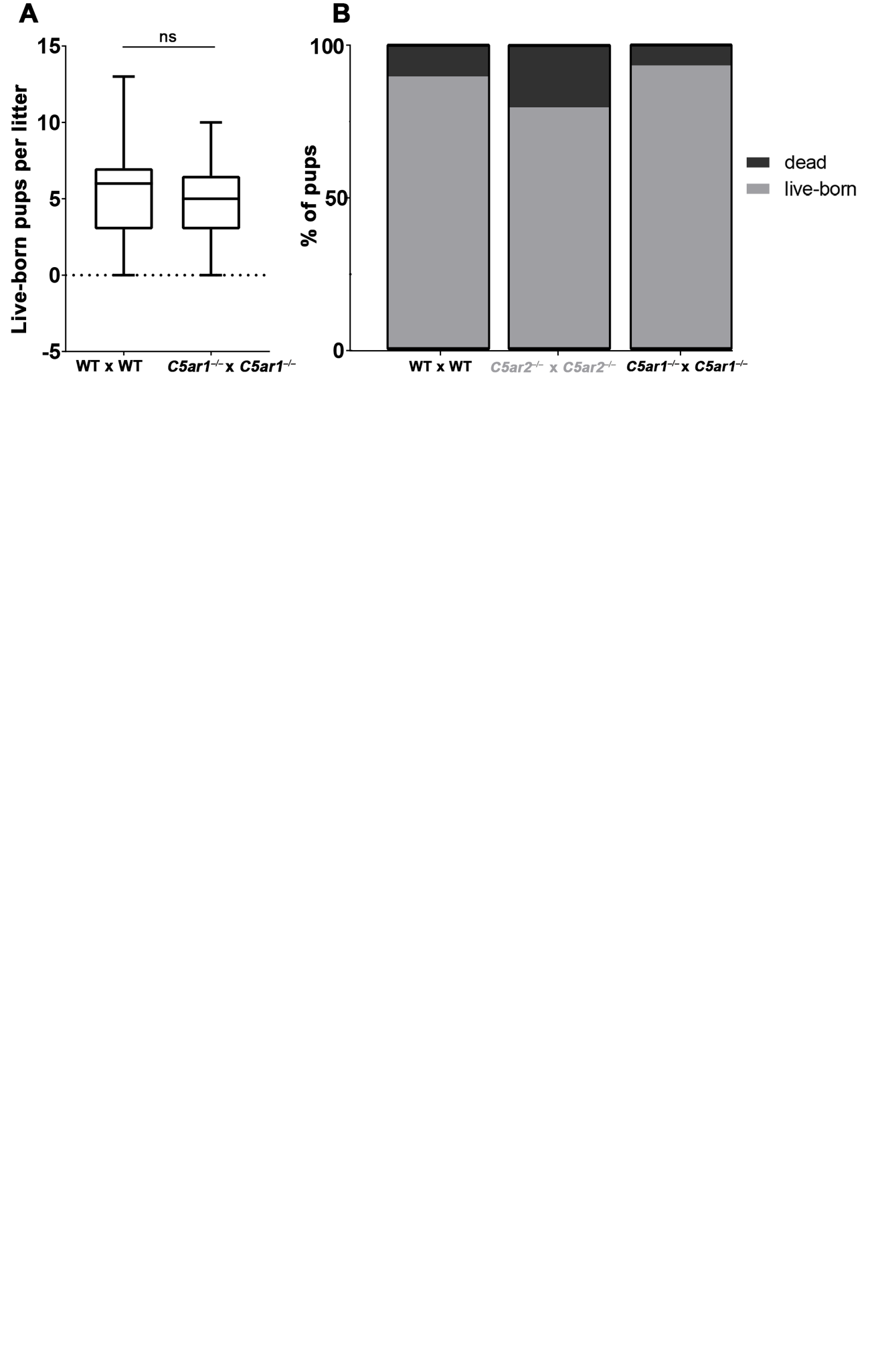
**

**Supplementary Figure S1: Deletion of *C5ar1* does not affect breeding efficiency.**

(A) Litter size of WT females mated with WT males (n = 98) compared to *C5ar1^–/–^* females mated with *C5ar1^–/–^* males (n = 37). Boxplots depict median and interquartile range; ns nonsignificant. (B) Ratios of live-born pups and pups found dead shortly after parturition in WT x WT (n = 590) compared to *C5ar2^–/–^* x *C5ar2^–/–^* (n = 259) and *C5ar1^–/–^* x *C5ar1^–/–^* (n = 191) matings. Ratios are given in %.


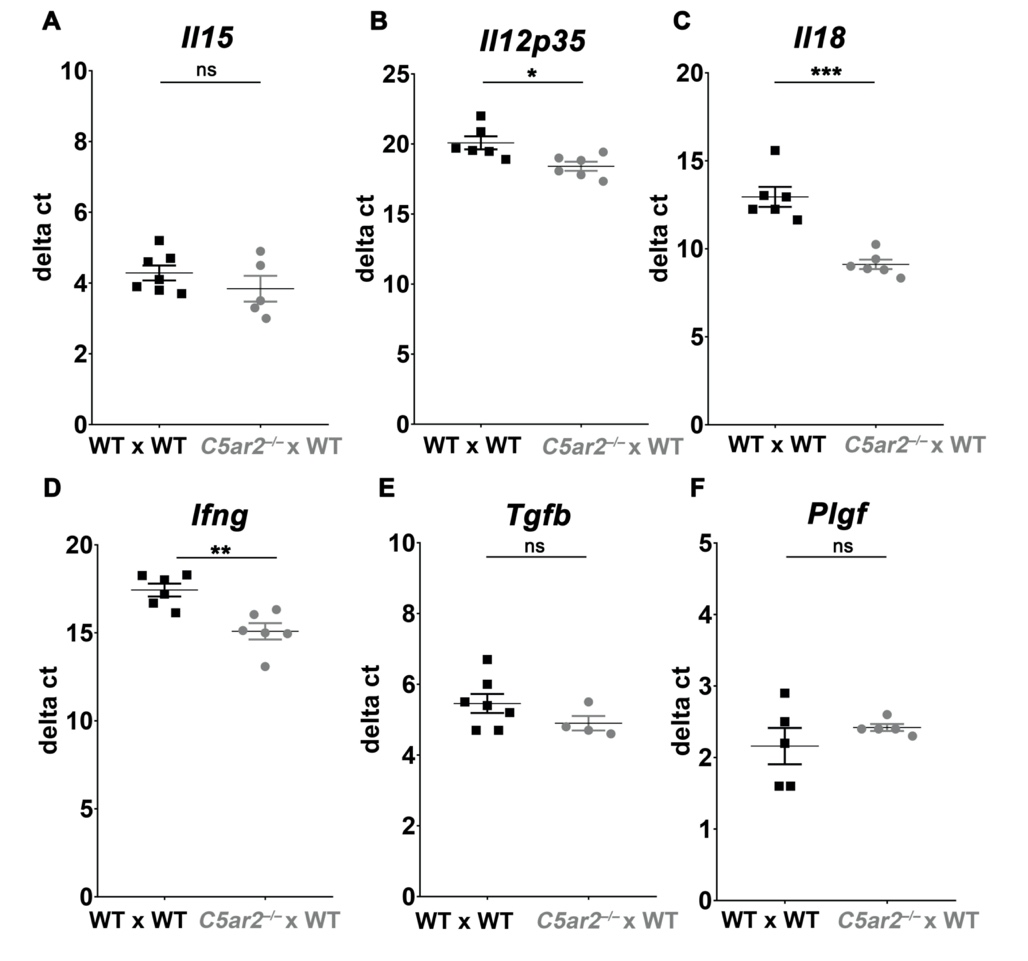


**Supplementary Figure S2:** **Statistical analysis of the mRNA levels of decidual cytokines**

Statistical analysis of the mRNA levels was performed by comparing the ΔCt values of *interleukin 15* (*Il15*), *interleukin 18* (*Il18*), *interferon gamma* (*Ifng*), and *transforming growth factor beta 1* (*Tgfb1*) and *placental growth factor* (*Plgf*) by unpaired T-test. Graphs depict mean and SEM. * p < 0.05; ** p < 0.01; *** p < 0.001; ns nonsignificant.


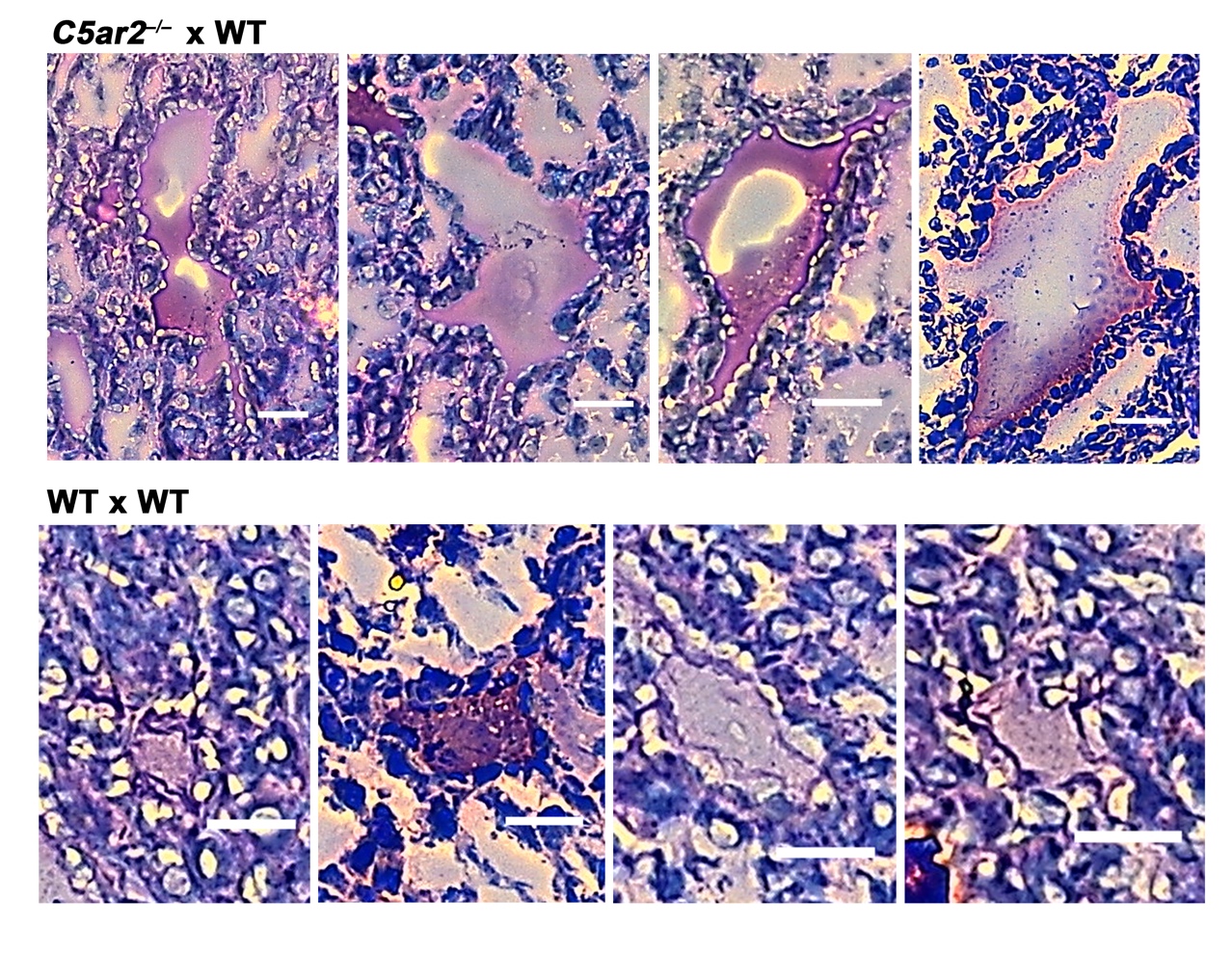


**Supplementary Figure S3:** **Hyperdilation of decidual vessels in IS of *C5ar2^–/–^* x WT matings**

Representative pictures of decidual vessels in IS of WT x WT (bottom panel) and *C5ar2^–/–^* x WT (top panel) matings at gd 10. The IS were stained with the Diff-Kwik staining kit. Bars indicate 50 µm.

**
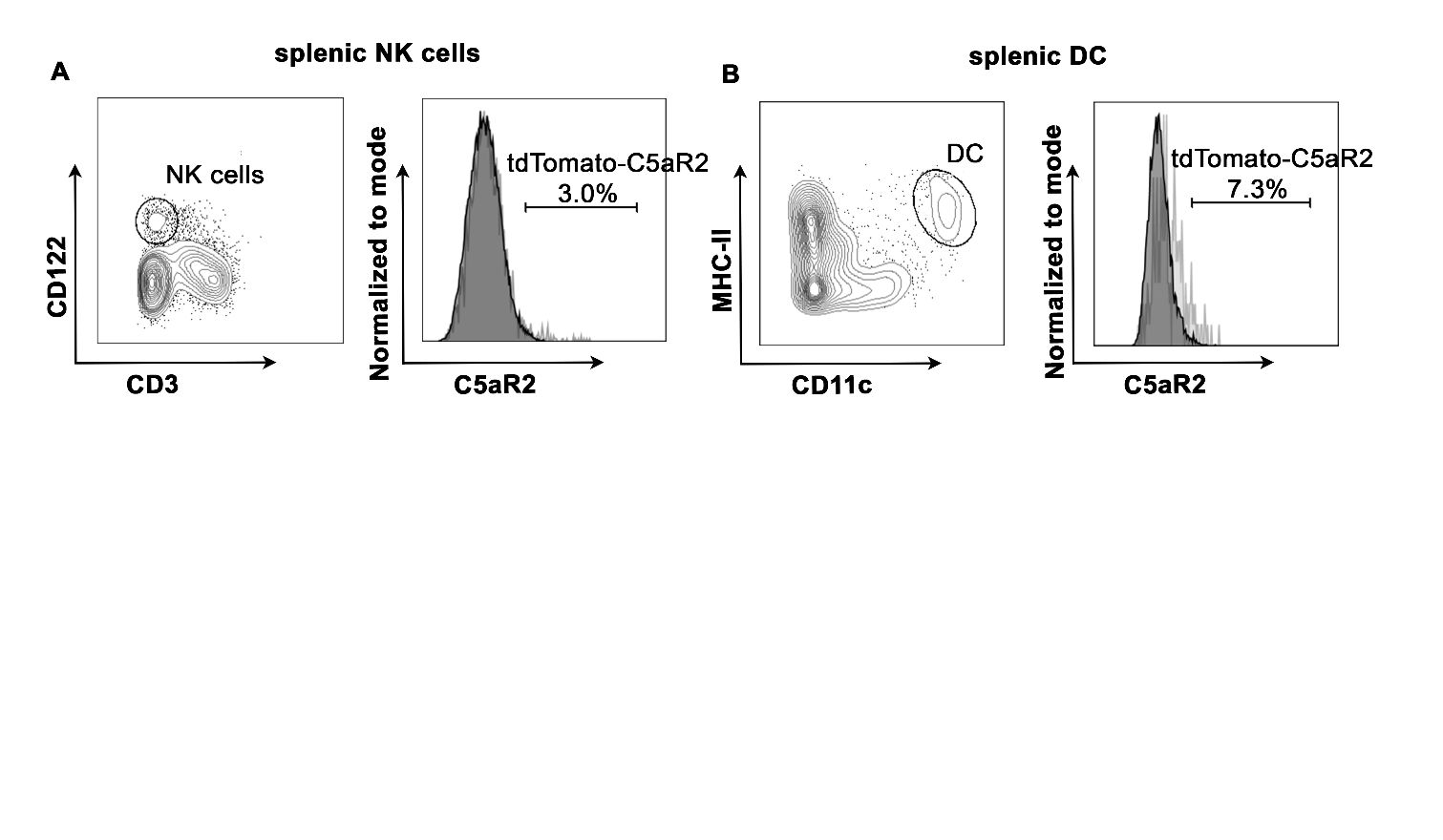
Supplementary Figure S4: C5aR2 expression in murine splenic NK cells and DC**

Representative FACS plots of (A) CD3^-^ CD122^+^ splenic NK cells and C5aR2-tdTomato signal of splenic NK cells from C5aR2-reporter x WT matings (light grey histogram) compared to WT x WT matings (dark grey histogram) at gd 8. Representative plots of (B) CD11c^+^MHC-II^+^ splenic DCs and C5aR2-tdTomato signal in splenic DC cells from C5aR2-reporter x WT matings (light grey histogram) compared to WT x WT matings (dark grey histogram) at gd8.


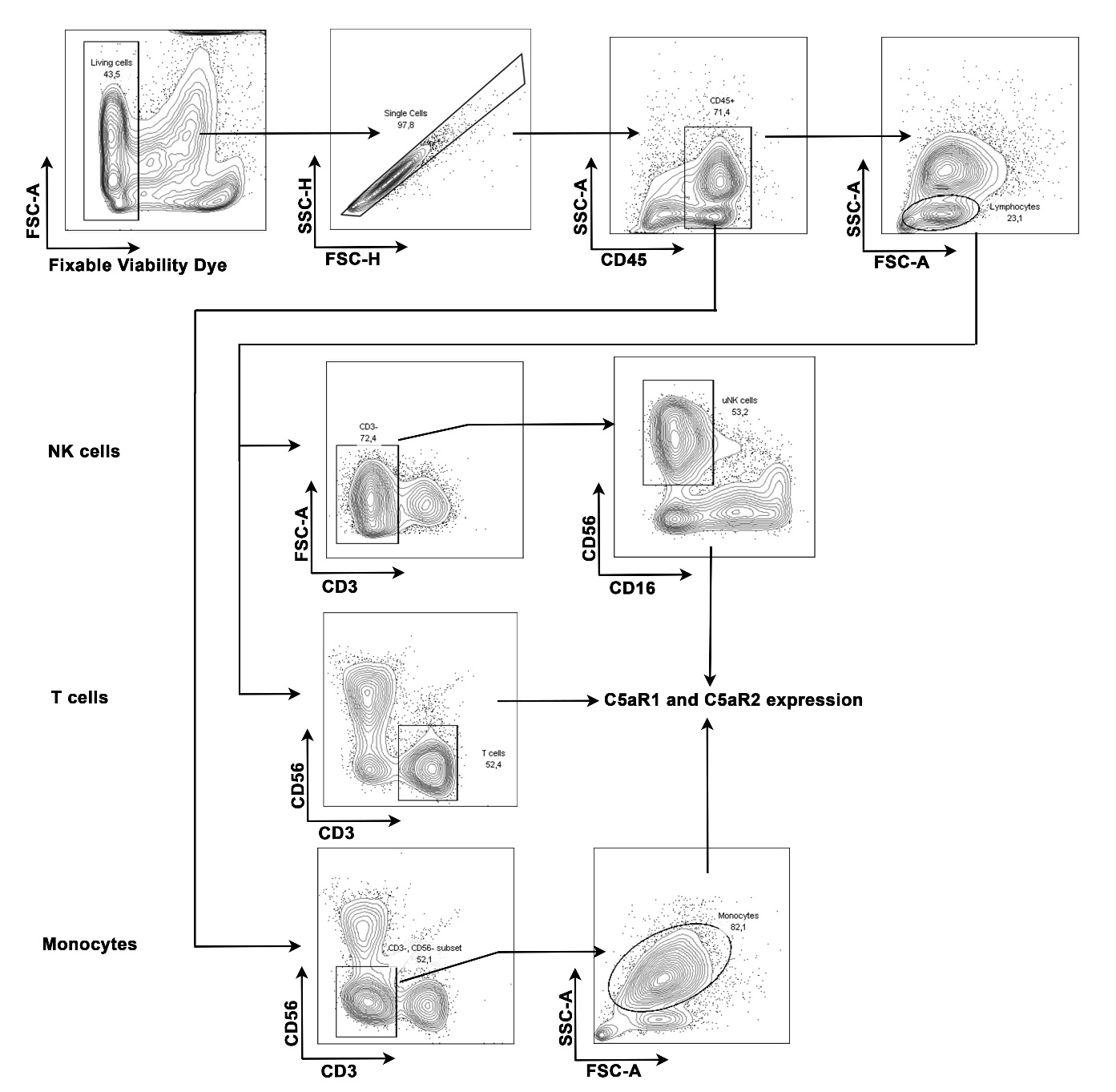


**Supplementary Figure S5: Gating strategy used for the analysis of human cells**

Gating strategy used for the flow cytometric analysis of human decidual and peripheral NK cells, T cells and MΦ/monocytes. Graphs depict the share of C5aR1^+^ or C5aR2^+^.

**
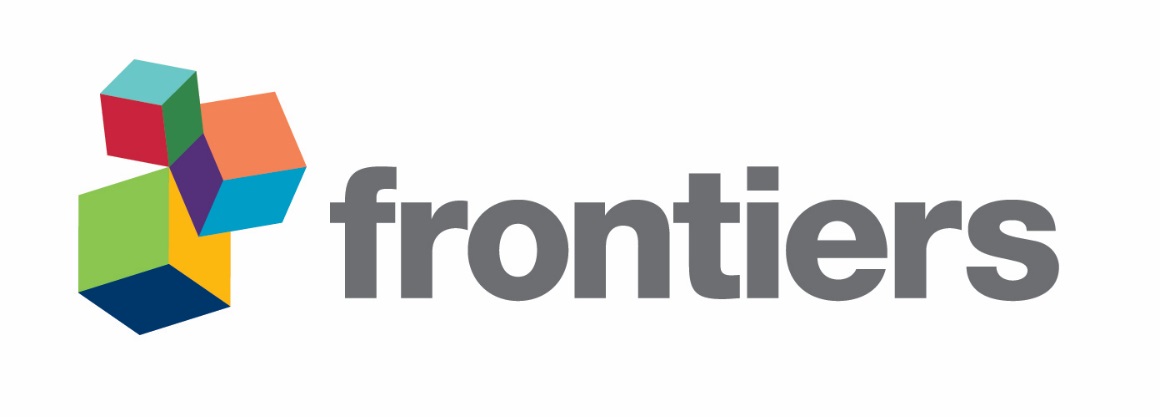
**
